# Effect of LDL cholesterol, statins and presence of mutations on the prevalence of type 2 diabetes in heterozygous familial hypercholesterolemia

**DOI:** 10.1038/s41598-017-06101-6

**Published:** 2017-07-17

**Authors:** Elisenda Climent, Sofía Pérez-Calahorra, Victoria Marco-Benedí, Nuria Plana, Rosa Sánchez, Emilio Ros, Juan F. Ascaso, Jose Puzo, Fátima Almagro, Carlos Lahoz, Fernando Civeira, Juan Pedro-Botet

**Affiliations:** 1 Lipid and Vascular Unit, Department of Endocrinology and Nutrition, Hospital del Mar, Universitat Autònoma de Barcelona, Barcelona, Spain; 2 Lipid Unit. Hospital Universitario Miguel Servet, IIS Aragón, Universidad de Zaragoza, Zaragoza, Spain; 3 Unitat de Medicina Vascular i Metabolisme, Hospital Universitari Sant Joan, Institut d´Investigació Sanitaria Pere Virgili (IISPV), Reus, Tarragona Spain; 4 Lipid Unit, Servicio de Endocrinología y Nutrición, Hospital Universitario Insular de Gran Canarias, Instituto Universitario de Investigaciones Biomédicas y Sanitarias de la Universidad de Las Palmas de Gran Canarias, Las Palmas, Spain; 5 Lipid Clinic, Endocrinology and Nutrition Service, Institut d’Investigacions Biomèdiques August Pi Sunyer, Hospital Clínic, Barcelona and CIBER Fisiopatología de la Obesidad y Nutrición (CIBEROBN), Instituto de Salud Carlos III (ISCIII), Madrid, Spain; 6 Servicio de Endocrinología y Nutrición, Hospital Clínico Universitario, Centro de Investigación Biomédica en Red de Diabetes y Enfermedades Metabólicas Asociadas (CIBERDEM), Universitat de Valencia, Valencia, Spain; 7 Lipid Unit. Hospital San Jorge, Huesca, Spain; 8 Lipid Unit, Hospital Donostia, San Sebastián, Spain; 9 Atherosclerosis Unit, Internal Medicine Department, Hospital Carlos III, Madrid, Spain

## Abstract

Patients with heterozygous familial hypercholesterolemia (HeFH) have been reported to be less vulnerable to type 2 diabetes mellitus (T2DM), although the mechanism is unknown. The aims of the present study were to assess the effects of low density lipoprotein (LDL) cholesterol concentration and the presence of FH-causing mutations on T2DM prevalence in HeFH. Data were collected from the Dyslipidemia Registry of the Spanish Arteriosclerosis Society. Inclusion criteria were definite or probable HeFH in patients aged ≥18 years. T2DM prevalence in HeFH patients was compared with data of the general population. 1732 patients were included. The prevalence of T2DM was lower in patients with HeFH compared with the general population (5.94% vs 9.44%; OR: 0.606, 95% CI 0.486–0.755, p < 0.001). Risk factors for developing T2DM were male sex, age, body mass index, hypertension, baseline triglyceride levels and years on statin therapy. The prevalence of T2DM in HeFH patients was 40% lower than that observed in the general population. Gene mutations and LDL cholesterol concentrations were not risk factors associated with the prevalence of T2DM in patients with HeFH. The prevalence of T2DM in patients with HeFH was 40% lower than in the general population matched for age and sex.

## Introduction

Heterozygous familial hypercholesterolemia (HeFH), the most frequent monogenic disorder of human metabolism caused by some mutations in the genes that encode for the low-density lipoprotein (LDL) receptor, apolipoprotein (apo) B, proprotein convertase subtilisin/kexin-type 9 (PCSK9) or apo E^1, 2^, entails an increased risk of premature cardiovascular disease^2^.

Patients with HeFH had been reported to be less vulnerable to type 2 diabetes mellitus (T2DM) compared to the general population^3^. More recently, Besseling *et al*. in a cross-sectional study found the prevalence of T2DM to be 50% lower in HeFH patients compared to unaffected relatives^4^. One of the mechanisms suggested to explain this finding lies in potential decreased cholesterol uptake by pancreatic β cells in HeFH^5^. This lower T2DM risk contrasts with the fact that most HeFH patients are treated with high doses of potent statins which exert a dose-dependent diabetogenic effect^6, 7^. The molecular pathways that may be involved in the resistance to T2DM in HeFH and the statin-related T2DM have not been studied in depth in humans. Suggested mechanisms include defects in insulin signaling, pancreatic β -cell function and reduced glucose uptake in adipocytes throughout the glucose transporter-4 (GLUT4) pathway^8^. The potential effect of LDLR mutations or LDL cholesterol concentration on these anti-diabetogenic and pro-diabetogenic effects of HeFH and statins, respectively, are unknown.

The cholesterol-lowering effect of statin therapy is mainly due to the inhibition of 3-hydroxy-3-methylglutaryl-CoA reductase, a key enzyme in cholesterol synthesis that induces a reduction in intracellular cholesterol content and a subsequent increase in LDL receptor expression in different tissues and the promotion of transmembrane cholesterol transport^9^, the primary altered mechanism in FH^10^. If intracellular cholesterol uptake via the LDL receptor is involved in the pathogenesis of T2DM, then it would explain the hypothetical protection from diabetes in HeFH patients, but only in those with genetic defects affecting LDL receptor uptake, in contrast to the hypothesis that the protection would be dependent on the high plasma LDL-cholesterol concentrations observed in HeFH. Hence, the aims of the present study were to assess the prevalence of T2DM in HeFH patients of the Dyslipidemia Registry of the Spanish Arteriosclerosis Society, and evaluate the impact of mutations in the LDLR, APOB and PCSK9 genes, baseline LDL cholesterol concentration and duration of statin treatment on T2DM prevalence in this population.

## Research Design and Methods

### Study population

The Dyslipidemia Registry of the Spanish Arteriosclerosis Society was created at the end of 2013 as an active on-line registry in which 50 certified Lipid Units distributed throughout Spain enter cases with different primary hyperlipidemias. These Lipid Units are the centers in the Spanish National Health Service to which most cases of primary hyperlipidemias are referred for diagnosis and treatment. The registry was approved by a central ethics committee to include anonymous clinical data (Comité Ético de Investigación Clínica de Aragón, Zaragoza, Spain) in accordance with the ethical guidelines of the 1975 Declaration of Helsinki. Patients did not have to complete an informed consent form as the data were obtained from an official national registry. Minimum data for the inclusion of cases in the registry are: age, sex, smoking status, personal history of hypertension, diabetes and cardiovascular disease with age at the first event, body mass index (BMI), waist circumference, total cholesterol, LDL cholesterol, triglycerides and high-density lipoprotein (HDL) cholesterol without lipid-lowering treatment, and clinical diagnosis. Cardiovascular disease in the registry is defined as: coronary heart disease (myocardial infarction, acute coronary syndrome with stenosis >50% of a main coronary artery and coronary revascularization), stroke (ischemic and hemorrhagic), aortic aneurysm and lower limb ischemia (intermittent claudication with ankle/brachial index <0.90 or revascularization of lower limb arteries). T2DM is defined in the presence of fasting blood glucose >125 mg/dL or use of blood glucose-lowering drugs.

Inclusion criteria in the present study were: complete minimum data for the registry, plus information on family history of hypercholesterolemia and premature cardiovascular disease; personal history of tendon xanthomas and presence of arcus cornealis before the age of 45; genetic study of LDLR, APOB and PCSK9 genes; age at statin onset; and age at T2DM diagnosis. Only subjects with probable (6–8 points) or definite (>8 points) HeFH according to the Dutch Lipid Clinic Network criteria^10^ and age ≥18 years were finally included. The diagnosis of homozygous FH (HoFH) was an exclusion criterion.

Of the 2,475 cases with the clinical diagnosis of genetic hypercholesterolemia included in the registry, 1,732 diagnosed of HeFH with Dutch Lipid Clinic Network (DLCN) criteria ≥6 points and complete data were finally included. Reasons for exclusion from the analysis were HoFH or DLCN <6 points and lack of data on genetic analysis, among others.

The prevalence of T2DM was analyzed according to a probable or definite diagnosis of HeFH, the presence of a genetic mutation or the gene involved. Furthermore, T2DM prevalence in HeFH patients was compared with data of the general population matched for age and sex from the Di@bet.es Study. The Di@bet.es Study is a national, cross-sectional, population-based survey conducted in 2009–10 in a representative random sample of the Spanish population^11^.

Blood specimens were collected after an overnight (>10 h) fast and processed for laboratory analyses the same day. Serum total cholesterol, triglycerides and HDL cholesterol levels were measured locally using enzymatic methods. Serum LDL cholesterol concentration was calculated using the Friedewald formula.

DNA was isolated from whole blood using standard methods and screening for LDLR, APOB and PCSK9 mutations was carried out using the Lipochip Platform (Progenika Biopharma S. A., Bilbao, Spain). The platform consists of two consecutive steps: the first is LIPOchip® microarray analysis for the detection of the most frequent Spanish point mutations in the LDLR gene and in the APOB exon 26, as well as CNVs in LDLR. When the LIPOchip® microarray yielded a negative result (no mutation is found), the LDLR, APOB (binding domain) and PCSK9 gene coding sequences, exon-intron boundaries, and short proximal intronic sequences were sequenced with a GS Junior system (Roche Diagnostics Corporation, Basel, Switzerland)^12^.

### Statistical analysis

Data were expressed as mean ± standard deviation for parametric variables and as median ± interquartile range for non-parametric quantitative variables. The Mann-Whitney U test was used for comparison between groups; quantitative variables were shown as % (n) and chi-square test was applied for qualitative variables between groups. Logistic regression analysis including sex, age, BMI, smoking status, hypertension, tendon xanthomas, baseline LDL and HDL cholesterol and triglyceride concentrations, genetic diagnosis and statin therapy duration was applied to evaluate factors independently associated with the presence of T2DM in HeFH patients. A two-sided p value < 0.05 was considered statistically significant. Statistical analysis was calculated with SPSS (version 19.0 for Windows; SPSS, Chicago, IL).

## Results

**Figure 1:**
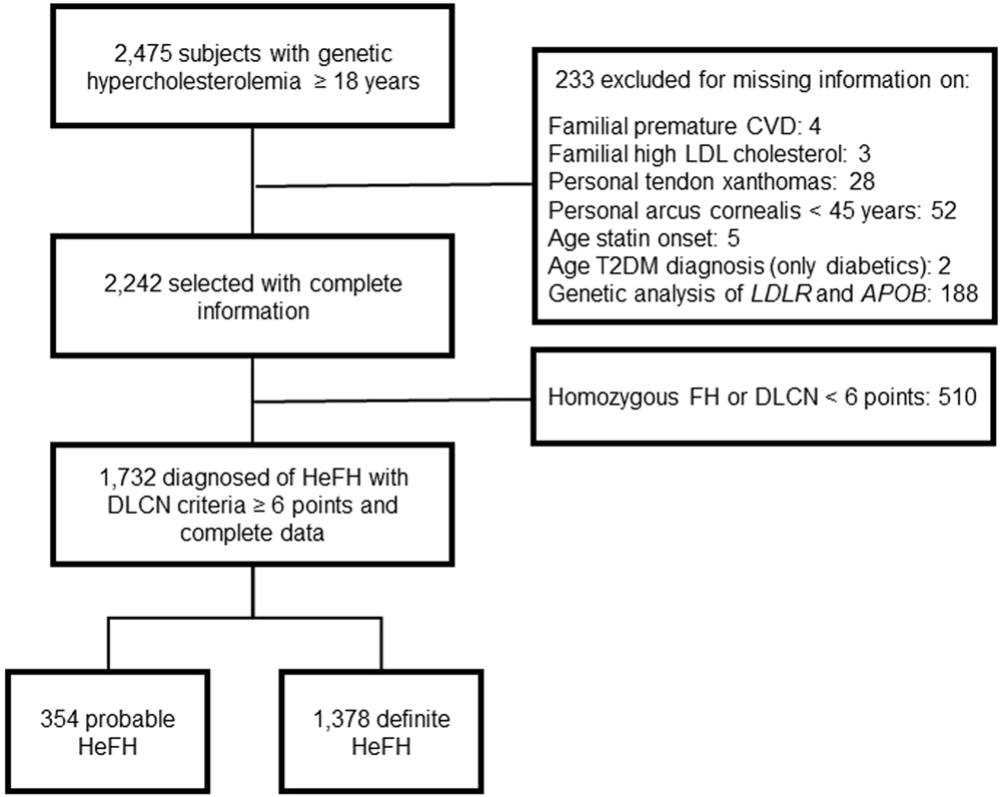
Study flow diagram. HeFH, heterozygous familial hypercholesterolemia; HoFH, homozygous familial hypercholesterolemia; DLCN, Dutch Lipid Clinic Network.

One thousand, seven hundred and thirty-two patients were included (354 with probable and 1,378 with definite HeFH) (Fig. 1). No differences were found between groups in terms of gender, cardiovascular disease prevalence and HDL cholesterol concentration. As expected because of the DLCN definition, total cholesterol and LDL cholesterol concentrations were higher, and the presence of tendon xanthomas more common in definite than in probable HeFH. In contrast, the prevalence of T2DM and variables associated with the metabolic syndrome and diabetes i.e. age, BMI, waist circumference, triglycerides, glucose and prevalence of hypertension were higher in the probable HeFH group than in definite HeFH. Subjects with definite HeFH had begun statin therapy 7 years earlier than probable HeFH (Table 1). A total of 103 (5.9%) HeFH were diagnosed of T2DM. The characteristics of subjects with and without T2DM are presented in Table 2. Diabetic patients were older and presented higher BMI, waist circumference, triglycerides, glycosylated hemoglobin, cardiovascular disease and hypertension prevalence than non-diabetics. HDL cholesterol was higher and a positive genetic diagnostic test was more common in non-diabetics than in diabetics. The number of years under statin treatment was higher in diabetics than in non-diabetics (Table 2).

**Table 1:** Demographic and clinical characteristics of heterozygous familial hypercholesterolemia patients.

Characteristics	Probable FH N = 354	Definite FH N = 1,378	*P*
Men, % (n)	50.0 (177)	48.9 (674)	0.715
Age, years	56.0 (49.0–64.5)	50.0 (38.0–60.0)	<0.001
Body mass index, kg/m^2^	26.7 (22.5–28.6)	26.2 (24.0–28.9)	0.006
Waist circumference, cm	93.0 (84.5–99.0)	90.0 (78.0–97.0)	<0.001
Total cholesterol, mg/dL	300 (272–333)	340 (298–395)	<0.001
Triglycerides, mg/dL	143 (99.5–194)	105 (75.0–143)	<0.001
HDL cholesterol, mg/dL	54.0 (46.5–64.5)	53.0 (44.0–64.0)	0.753
LDL cholesterol, mg/dL	213 (190–251)	260 (216–316)	<0.001
Glucose, mg/dL	91.0 (84.5–97.5)	89.0 (81.0–98.0)	0.051
Tendon xanthomas, % (n)	3.3 (12)	35.2 (485)	<0.001
Cardiovascular disease, % (n)	11.9 (42)	13.4 (185)	0.489
Hypertension, % (n)	26.4 (93)	17.7 (244)	<0.001
Diabetes, % (n)	9.1 (32)	5.2 (71)	0.006
Age at diabetes diagnosis, years	54.0 (50.8–60.0)	52.5 (46.5–60.3)	0.563
Age at statin onset, years	48.3 (37.0–56.0)	41.0 (31.0–51.0)	<0.001
Statin therapy, years	5.0 (2.0–11.0)	7.0 (3.0–13.0)	<0.001

Values are expressed as median (interquartile range) for continuous variables and percentage (n) for categorical variables.

HDL denotes high-density lipoprotein; LDL, low-density lipoprotein.

**Table 2:** Demographic and clinical characteristics of heterozygous familial hypercholesterolemia patients with or without diabetes.

Characteristics	Diabetes N = 103	No diabetes N = 1,629	*P*
Men, % (n)	58.3 (60)	48.6 (792)	0.057
Age, years	64 (55.0–69.0)	51.0 (40.0–60.0)	<0.001
Body mass index, kg/m^2^	28.9 (25.3–33.3)	25.5 (22.8–28.4)	<0.001
Waist circumference, cm	100 (92.0–108)	89.0 (80.0–96.0)	<0.001
Total cholesterol, mg/dL	348 (291–395)	331 (292–384)	0.318
Triglycerides, mg/dL	189 (129–256)	106 (78.0–155)	<0.001
HDL cholesterol, mg/dL	47.5 (35.0–59.0)	53.0 (45.0–64.0)	<0.001
LDL cholesterol, mg/dL	255 (202–312)	253 (211–300)	0.960
HbA1c*, %	6.7 (6.2–7.4)	5.5 (5.3–5.9)	<0.001
Tendon xanthomas, % (n)	32.0 (33)	28.7 (468)	0.487
Positive genetic diagnosis, % (n)	46.6 (48)	64.6 (1052)	0.005
Cardiovascular disease, % (n)	40.8 (42)	11.3 (184)	<0.001
Hypertension, % (n)	64.1 (66)	16.7 (271)	<0.001
Age at diabetes diagnosis, years	53.0 (48.0–60–0)		—
Age at statin onset, years	51.5 (43.0–59.0)	42.0 (31.5–52.0)	<0.001
Statin therapy, years	10.0 (6.0–17.0)	7.0 (2.5–12.0)	<0.001

*Values refer to 87 (84.5%) diabetics and 410 (25.2%) non-diabetics.

**Figure 2:**
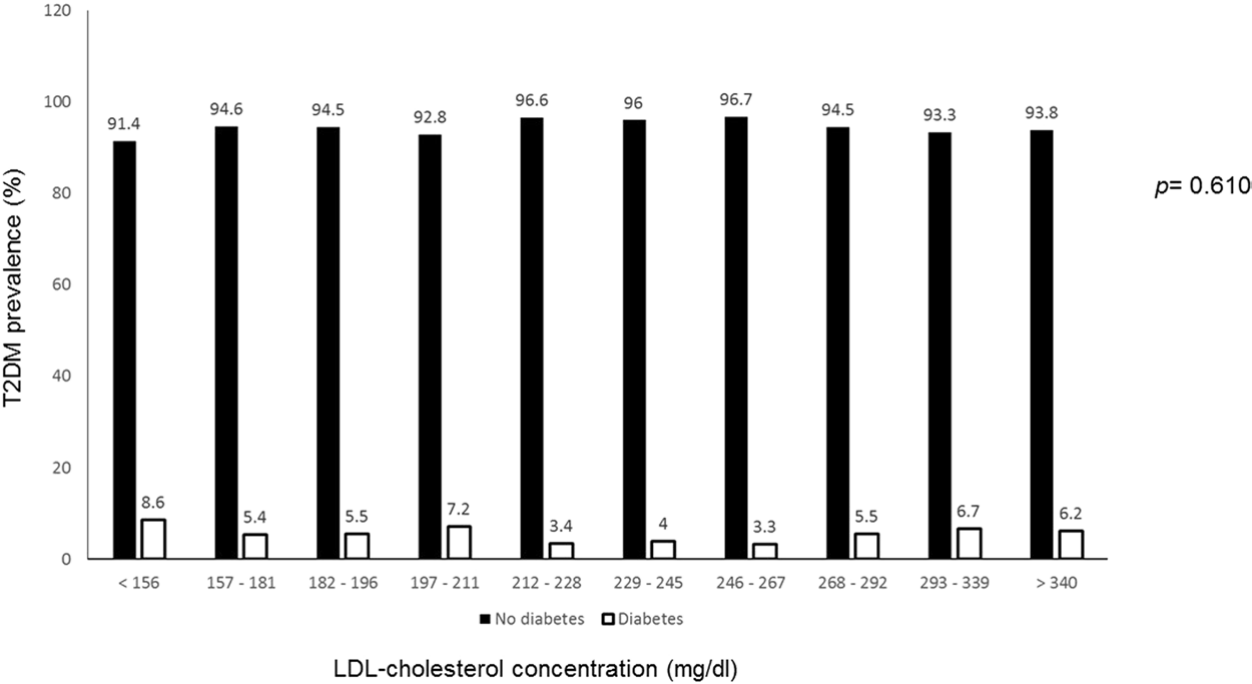
Prevalence of type 2 diabetes mellitus in heterozygous familial hypercholesterolemic patients according to LDL-cholesterol concentrations.

The overall and divided-by-age group gender and genetic diagnosis prevalence of T2DM in HeFH with the odds ratios (OR) compared with the prevalence of the age- and gender-adjusted Spanish population are shown in Table 3. T2DM was more common in males than in females and increased with age in HeFH and in the general population. However, T2DM was significantly less frequent among HeFH, 5.94% versus 9.44% in the general population (OR: 0.606, 95% CI 486–0.755, p < 0.001). This difference was also present when only HeFH with a positive genetic diagnosis (OR: 0.438, 95% CI 0.323–0.593, p < 0.001) were considered.T2DM protection in HeFH was similar in all age groups and in both genders, but reached statistical significance only in older groups, owing to the low T2DM prevalence in subjects under 46 years of age (Table 3). Subjects with a functional mutation in candidate genes were older, had higher BMI, waist circumference, triglyceride and glucose concentrations, and a higher prevalence of hypertension and T2DM than HeFH without mutations (Supplementary Table). On the other hand, no differences were observed in the prevalence of T2DM when patients were separated into different subgroups according to LDL-cholesterol concentrations (Fig. 2).

**Table 3:** Prevalence of type 2 diabetes in a reference population and in heterozygous familial hypercholesterolemia patients by sex and age.

	18–45 years	46–60 years	>60 years	Total
**Men**				
General population, N/Total (%)	14/909 (1.54)	69/578 (11.9)	163/687 (23.7)	246/2174 (11.3)
HeFH, N/Total (%)	2/337 (0.59)	23/338 (6.80)	35/178 (19.7)	60/853 (7.03)
Odds ratio (95% CI)	0.382 (0.086–1.688)	0.538 (0.329–0.881)	0.787 (0.523–1.185)	0.593 (0.442–0.796)
P	0.261	0.012	0.272	<0.001
HeFH mutation+, N/Total (%)	2/246 (0.81)	15/188 (7.98)	13/103 (12.6)	30/537 (5.59)
Odds ratio (95% CI)	0.524 (0.118–2.231)	0.639 (0.356–1.147)	0.478 (0.263–0.870)	0.470 (0.319–0.694)
P	0.545	0.141	0.010	<0.001
**Women**				
General population, N/Total (%)	9/1222 (0.74)	54/818 (6.60)	170/858 (19.8)	233/2898 (8.04)
HeFH, N /Total (%)	2/277 (0.72)	18/318 (5.66)	23/285 (8.07)	43/880 (4.89)
Odds ratio (95% CI)	0.980 (0.211–4.562)	0.849 (0.489–1.471)	0.355 (0.225–0.562)	0.588 (0.421–0.821)
P	0.979	0.684	<0.001	0.002
HeFH mutation+, N/Total (%)	2/221 (0.90)	6/185 (3.24)	10/157 (6.37)	18/563 (3.20)
Odds ratio (95% CI)	1.455 (0.359–5.093)	0.508 (0.222–1.164)	0.287 (0.150–0.549)	0.387 (0.239–0.627)
P	0.597	0.087	<0.001	<0.001
**All**				
General population, N/Total (%)	23/2131 (1.08)	123/1396 (8.81)	333/1545 (21.6)	479/5072 (9.44)
HeFH, N /Total (%)	4/614 (0.65)	41/656 (6.25)	58/463 (12.5)	103/1733 (5.94)
Odds ratio (95% CI)	0.601 (0.207–1.744)	0.689 (0.478–0.995)	0.521 (0.386–0.704)	0.606 (0.486–0.755)
P	0.343	0.046	<0.001	<0.001
HeFH mutation+, N/Total (%)	4/467 (0.86)	21/373 (5.63)	23/260 (8.85)	48/1100 (4.36)
Odds ratio (95% CI)	0.791 (0.273–2.300)	0.617 (0.383–0.995)	0.353 (0.226–0.551)	0.438 (0.323–0.593)
P	0.667	0.046	<0.001	<0.001

Risk factors for HeFH patients developing T2DM are presented in Table 4 for the whole HeFH group and in Table 5 for patients with a positive genetic diagnosis. Male sex, BMI, hypertension and years on statin therapy were independently associated with the presence of T2DM in both groups. These risk factors could account for up to 26% of the variability in presenting T2DM (Table 4). Other factors such as age, BMI, smoking status, xanthomas, baseline LDL, HDL cholesterol and triglyceride concentrations did not reach statistical significance.

**Table 4:** Factors associated with the presence of type 2 diabetes mellitus in clinically-defined heterozygous familial hypercholesterolemia patients (≥6 DLCN points).

	Standard coeff. (β)	Exp (B)	95% CI	*P*	R^2^
Sex, male	0.649	1.913	1.204–3.040	0.006	0.261
Age, years	0.050	1.052	1.030–1.074	<0.001	
BMI, kg/m^2^	0.021	1.021	1.007–1.035	0.004	
Hypertension, yes	1.522	4.582	2.834–7.407	<0.001	
Triglycerides, mg/dL	0.002	1.002	1.001–1.003	0.001	
Statin therapy, years	0.031	1.031	1.003–1.060	0.03	

DLCN denotes Dutch Lipid Clinic Network; BMI, body mass index.

Independent variables included in the model: sex, age, BMI, smoking status, hypertension, tendon xanthomas, baseline LDL and HDL cholesterol and triglyceride concentrations, genetic diagnosis and statin therapy duration.

**Table 5:** Factors associated with the presence of type 2 diabetes mellitus in genetically-defined heterozygous familial hypercholesterolemia patients (with a pathogenic mutation in *LDLR*, *APOB* or *PCSK9 genes*).

	Standard coeff. (β)	Exp (B)	95% CI	*p*	R^2^
Sex, male	0.713	2.041	1.081–3.853	0.028	0.195
BMI, kg/m^2^	0.018	1.018	1.003–1.034	0.022	
Hypertension, yes	2.072	7.942	4.249–14.845	<0.001	
Statin therapy, years	0.044	1.045	1.002–1.090	0.040	

BMI denotes body mass index.

Independent variables included in the model: sex, age, BMI, smoking status, hypertension, tendon xanthomas, baseline LDL and HDL cholesterol and triglyceride concentrations, genetic diagnosis and statin therapy duration.

Finally, different regression models were applied to identify the association of a pathogenic mutation in *LDLR*, *APOB* or *PCSK9* in the presence of T2DM. None of the models with different adjustments showed any significant association of a positive genetic diagnosis with T2DM (Table 6).

**Table 6:** Effect of phenotype severity in heterozygous familial hypercholesterolemia on type 2 diabetes prevalence.

Comparison	All	HeFH with LDL <250 mg/dL	HeFH with LDL ≥250 mg/dL	HeFH with >8 points
Odds ratio (95% CI) Unadjusted	0.821 (0.429–1.572)	0.736 (0.287–1.887)	2.384 (0.557–10.207)	0.707 (0.294–1.696)
Odds ratio (95% CI) Adjusted^*^	0.954 (0.488–1.866)	1.386 (0.498–3.852)	2.395 (0.549–10.444)	0.747 (0.303–1.839)
Odds ratio (95% CI) Adjusted^†^	0.936 (0.478–1.835)	1.388 (0.496–3.887)	2.806 (0.631–12.478)	0.735 (0.298–1.813)
Odds ratio (95% CI) Adjusted^‡^	1.072 (0.533–2.155)	1.474 (0.512–4.243)	3.056 (0.670–13.930)	0.877 (0.341–2.250)

^*^Adjusted for age, sex and years of statin use.

^†^Adjusted for age, sex, years of statin use and body mass index.

^‡^Adjusted for age, sex, years of statin use, body mass index, triglycerides, and hypertension.

## Discussion

The prevalence of T2DM in HeFH patients in the present study was 5.9%, lower than the known 9.4% prevalence in the age and sex-adjusted Spanish population^11^. In a Dutch cohort, the prevalence of T2DM in HeFH patients was 1.75% compared to 2.93% in unaffected relatives^4^. This may be partially explained by younger age in the Dutch HeFH cohort, more than one decade, compared to our population and the different criteria used in the diagnosis of T2DM between studies, only auto-reported in the Dutch cohort^4^. Nevertheless, our study confirms the Dutch results in a completely different population, showing that HeFH subjects present an approximately 50% lower T2DM risk than the general population. This T2DM protection has been questioned in favor of a role for cellular cholesterol metabolism in the pathogenesis of type 2 diabetes^4, 5^.

Besides this confirmatory information, several important conclusions can be drawn from our study. First, the LDL cholesterol concentration before the start of lipid-lowering treatment did not play a major role in T2DM protection in HeFH in our study. The lower risk of T2DM appeared in a large LDL cholesterol range, and the LDL cholesterol concentration did not significantly affect T2DM risk. Although there is *in-vivo* and *ex-vivo* evidence showing that LDL concentration modulates insulin secretion and pancreatic β-cell survival^13^, large prospective studies showed no significant effect of LDL cholesterol on T2DM risk, in contrast to the association with high triglycerides or low HDL cholesterol that has been repeatedly demonstrated^14, 15^. In human β cell islets, LDL overload lowered insulin secretion^16^ and diet-induced hypercholesterolemia also reduced insulin secretion in mice^17^; however, this diabetogenic effect of hypercholesterolemic diets has also been associated with saturated fatty acid accumulation rather than with cholesterol^16, 18^. Second, in the present study, no differences were observed in T2DM prevalence according to the presence or absence of causative gene mutations responsible for HeFH. Our study is the first to include clinically-diagnosed patients and analyzed T2DM prevalence according to *LDLR*, *APOB* or *PCSK9* mutations. Besseling *et al*. included HeFH only with a confirmed pathogenic mutation, and found a dose-dependent association in HeFH subjects with *LDLR* negative mutations who had a lower T2DM prevalence than HeFH carriers of defective *LDLR* or *APOB* mutations, suggesting that the severity of the genetic defect plays a role in T2DM protection^4^. The implication of the LDL receptor in glucose homeostasis is controversial, since the deleterious effect of cholesterol overload on insulin secretion by pancreatic β cells was similar in LDLR-/- knockout or wild-type mice^19^, and oxidized LDL, which does not use the LDL receptor, was found to be a major contributor to β cell dysfunction^20^. Furthermore, some FH-causing mutations seem to be associated with a higher T2DM risk^21^. In our study, the presence of any LDLR/APOB/PCSK9 mutation was less common in HeFH with than without T2DM; however, these differences disappeared after adjustment for confounders unrelated to LDL receptor function.

Third, the factors associated with the presence of T2DM in HeFH are the same as those present in the general population, including statin use. The cluster of abnormalities closely associated with insulin resistance: high blood pressure, high triglycerides, low HDL cholesterol, increased BMI and older age^22^, are also strongly related to the presence of T2DM in HeFH, as previously described^23^. In this observational, cross-sectional study, we could not calculate whether the impact of T2DM risk factors is different in HeFH than in non-FH; however, we did have a close approximation for statin use as this information was available. Statin use preceded the diagnosis of T2DM in most cases and the risk associated with statin use was identical to that observed in statin trials after adjustment for insulin-resistance markers^6, 7, 24^.

Thus, if LDL cholesterol or LDL receptor function itself are not responsible for the T2DM protection in HeFH, which mechanisms are involved? Potential explanations have been previously put forward: the observational cross-sectional design without power to establish causality; the possibility of unexplored potential confounders; a healthier lifestyle after receiving the diagnosis of HeFH; or a shorter life-expectancy in patients with HeFH and T2DM^4^ could be responsible for this observation. The HDL particle is also an unexplored potential protective factor of T2DM in HeFH^25^. In HeFH, HDL cholesterol is usually within normal range^10, 26^ and, in contrast with other dyslipidemias, HDL particle size and number are in antiatherogenic range^27^. Indeed, the Dutch HeFH patients were leaner, smoked less and had lower triglycerides than their unaffected relatives without a clear explanation, except for a healthier lifestyle associated with the diagnosis^4^. In our cohort of HeFH patients, the prevalence of hypertension, overweight, obesity and tobacco use was much lower than that observed in the Spanish general population^28, 29^. Altogether, these data suggest that environmental factors could be involved in this T2DM protection in these patients cared for in highly specialized lipid units. Actually, the reduction in BMI compared with the reference population could explain one third of the lower risk of T2DM^30^. A large case-control study is underway to try to answer these questions.

Our study has some limitations. In this respect, the number of HeFH with T2DM was relatively low, but the concurrence with previous data supports the T2DM protection. Our study was cross-sectional, and results other than prevalence should thus be taken with caution. The sample was composed of middle-aged men and women from a Mediterranean country, with traditionally lower coronary heart disease rates than other populations, albeit with a high prevalence of T2DM^11^. Furthermore, patients included in the Spanish Dyslipidemia Registry are treated with a standardized protocol promoting the use of high-intensity lipid-lowering treatment in highly specialized Lipid Clinics; thus, the results cannot be easily extrapolated to other clinical settings. Finally, the observed prevalence of T2DM in HeFH could be underestimated since patients with HeFH are at increased risk for cardiovascular disease and some will have died before their inclusion in the Dyslipidemia Spanish registry. If HeFH was associated with T2DM, the possibility of early death from cardiovascular causes was even higher. On the other hand, T2DM prevalence increases with age and HeFH patients who died prematurely might have been less likely to develop T2DM; thus, an overestimation of T2DM in our cohort is also possible. In any event, the mean age of HeFH patients was around 55 years, and the prevalence of T2DM increased with age in both HeFH patients and in the general population.

## Conclusions

In summary, the absolute prevalence of T2DM in patients with HeFH was 40% lower than that observed in the general population matched for age and sex. Risk factors independently associated with the presence of T2DM in subjects with HeFH were age, male sex, BMI, baseline triglycerides, hypertension and years of statin treatment. LDL cholesterol concentrations and the presence of mutations in FH-causing genes were not associated with the presence of T2DM in HeFH subjects.

## Electronic supplementary material

Supplementary Information
