# Supplementary material for: Effect of LDL cholesterol, statins and presence of mutations on the prevalence of type 2 diabetes in heterozygous familial hypercholesterolemia
Source: Sci Rep. 2017 Jul 17;7:5596. doi: 10.1038/s41598-017-06101-6 (PMC5514105; doi:10.1038/s41598-017-06101-6)
Supplement: Supplementary file 1 — Supplementary Information [file 41598_2017_6101_MOESM1_ESM.doc]

**TITLE PAGE**

Effect of LDL cholesterol, statins and presence of mutations on the prevalence of type 2 diabetes in heterozygous familial hypercholesterolemia

**Authors:**

Elisenda Climent, MD^a^, Sofía Pérez-Calahorra, RD, MSc^b^, Victoria Marco-Benedí, RD^b^, Nuria Plana, MD, PhD^c^, Rosa Sánchez, MD^d^, Emilio Ros, MD, PhD^e^, Juan F Ascaso, MD, PhD^f^, Jose Puzo, MD, PhD^g^, Fátima Almagro, MD,

PhD^h^, Carlos Lahoz, MD, PhD^i^, Fernando Civeira, MD, PhD^b^, Juan Pedro-Botet, MD, PhD^a*^

^a^Lipid and Vascular Unit, Department of Endocrinology and Nutrition, Hospital del Mar, Universitat Autònoma de Barcelona, Barcelona, Spain.

^b^Lipid Unit. Hospital Universitario Miguel Servet, IIS Aragón, Universidad de Zaragoza, Zaragoza, Spain.

^c^Unitat de Medicina Vascular i Metabolisme, Hospital Universitari Sant Joan, Institut d´Investigació Sanitaria Pere Virgili (IISPV), Reus, Tarragona, Spain. ^d^Lipid Unit, Servicio de Endocrinología y Nutrición. Hospital Universitario Insular de Gran Canarias, Instituto Universitario de Investigaciones Biomédicas y Sanitarias de la Universidad de Las Palmas de Gran Canarias, Las Palmas, Spain.

^e^Lipid Clinic, Endocrinology and Nutrition Service, Institut d’Investigacions Biomèdiques August Pi Sunyer, Hospital Clínic, Barcelona and CIBER Fisiopatología de la Obesidad y Nutrición (CIBEROBN), Instituto de Salud Carlos III (ISCIII), Spain.

^f^Servicio de Endocrinología y Nutrición, Hospital Clínico Universitario, Centro de Investigación Biomédica en Red de Diabetes y Enfermedades Metabólicas Asociadas (CIBERDEM), Universitat de Valencia, Valencia, Spain.

^g^Lipid Unit. Hospital San Jorge, Huesca, Spain.

^h^Lipid Unit, Hospital Donostia, San Sebastián, Spain.

^i^Atherosclerosis Unit, Internal Medicine Department, Hospital Carlos III, Madrid, Spain.

**CORRESPONDENCE TO:**

Dr. Juan Pedro-Botet

Department of Endocrinology

Hospital del Mar; Paseo Marítimo, 25-29; E-08003 Barcelona, Spain

Phone: 34-932483902. FAX: 34-932483254. Email: [86620@parcdesalutmar.cat](mailto:86620@parcdesalutmar.cat)

Supplementary Table. Demographic and clinical characteristics of heterozygous familial hypercholesterolemia patients with or without a pathogenic mutation in *LDLR*, *APOB* or *PCSK9* genes.

| Characteristics | HeFH mutation +  n = 1,100 | HeFH mutation -n = 578 | *P* |
| --- | --- | --- | --- |
| Men, % (n) | 48.8 (537) | 48.1 (278) | 0.779 |
| Age, years | 49.5 (37.0-60.0) | 55.0 (46.0-63.0) | <0.001 |
| Body mass index, kg/m^2^ | 25.2 (22.3-28.3) | 26.4 (24.1-29.2) | <0.001 |
| Waist circumference, cm | 88.0 (78.0-95.0) | 91.0 (84.0-98.5) | <0.001 |
| Total cholesterol, mg/dL | 334 (290-389) | 332 (297-377) | 0.831 |
| Triglycerides, mg/dL | 98.0 (70.0-139) | 130 (92.0-189) | <0.001 |
| HDL cholesterol, mg/dL | 52.0 (44.0-63.0) | 54.0 (46.0-64.0) | 0.025 |
| LDL cholesterol, mg/dL | 257 (212-313) | 250 (208-287) | 0.012 |
| Glucose, mg/dL | 88.0 (81.0-97.0) | 92.0 (84.0-99.8) | 0.001 |
| Tendon xanthomas, % (n) | 30.9 (330) | 27.0 (150) | 0.100 |
| Cardiovascular disease, % (n) | 12.8 (141) | 12.5 (72) | 0.868 |
| Hypertension, % (n) | 14.9 (164) | 26.5 (153) | <0.001 |
| Diabetes, % (n) | 4.4 (48) | 7.4 (43) | 0.008 |
| Age at diabetes diagnosis, years | 51.0 (43.8-60.3) | 53.0 (51.0-60.0) | 0.302 |
| Age at statin onset, years | 40.0 29.0-50.0) | 46.0 (37.0-55.0) | <0.001 |
| Statin therapy, years | 8.0 (3.0-13.0) | 6.0 (2.0-12.0) | 0.001 |

HDL denotes high-density lipoprotein; LDL, low-density lipoprotein.
